# Biomarkers and polymorphisms in pancreatic neuroendocrine tumors treated with sunitinib

**DOI:** 10.18632/oncotarget.26380

**Published:** 2018-12-11

**Authors:** Paula Jiménez-Fonseca, Miguel Navarro Martín, Alberto Carmona-Bayonas, Alfonso Calvo, Javier Fernández-Mateos, Miriam Redrado, Jaume Capdevila, Nieves Martínez Lago, Adelaida Lacasta, Javier Muñarriz, Ángel Segura, Josep Fuster, Francisco Barón, Marta Llanos, Raquel Serrano, Alfredo Castillo, Juan Jesús Cruz Hernández, Enrique Grande

**Affiliations:** ^1^ Medical Oncology Department, Hospital Universitario Central de Asturias, Oviedo, Spain; ^2^ Medical Oncology Department, Hospital Universitario de Salamanca, IBSAL, Salamanca, Spain; ^3^ Hematology and Medical Oncology Department, Hospital Universitario Morales Meseguer, UMU, IMIB, Murcia, Spain; ^4^ IDISNA and Program in Solid Tumors and Biomarkers, Center for Applied Medical Research (CIMA), Department of Histology and Pathology, University of Navarra, CIBERONC, ISC-II, Pamplona, Spain; ^5^ Molecular Medicine Unit, IBSAL, Department of Medicine, University of Salamanca, Salamanca, Spain; ^6^ IDISNA and Program in Solid Tumors and Biomarkers, Center for Applied Medical Research (CIMA), Department of Histology and Pathology, University of Navarra, Pamplona, Navarra, Spain; ^7^ Medical Oncology Department, Hospital Universitario Vall d’Hebron, Autonomous University of Barcelona, Barcelona, Spain; ^8^ Medical Oncology Department, Hospital Universitario de A Coruña, La Coruña, Spain; ^9^ Medical Oncology Department, Hospital Universitario Donostia, Guipúzcoa, Spain; ^10^ Medical Oncology Department, Hospital General Universitario de Castellón, Castellón, Spain; ^11^ Medical Oncology Department, Hospital Universitario La Fe, Valencia, Spain; ^12^ Medical Oncology Department, Hospital Universitario Son Espases, Palma de Mallorca, Spain; ^13^ Medical Oncology Department, Hospital Universitario de Santiago de Compostela, Santiago de Compostela, Spain; ^14^ Medical Oncology Department, Hospital Universitario de Canarias, Santa Cruz de Tenerife, Spain; ^15^ Medical Oncology Department, Hospital Universitario Reina Sofia, Cordoba, Spain; ^16^ Medical Oncology Department, MD Anderson Cancer Center, Madrid, Spain

**Keywords:** sunitinib, osteopontin, IL-6, VEGFR-3, pancreatic neuroendocrine tumors

## Abstract

Several circulating biomarkers and single nucleotide polymorphisms (SNPs) have been correlated with efficacy and tolerability to antiangiogenic agents. These associations remain unexplored in well-differentiated, metastatic pancreatic neuroendocrine tumors treated with the multitargeted tyrosine kinase inhibitor sunitinib. We have assessed the effect on tumor response at 6 months, overall survival, progression-free survival and safety of 14 SNPs, and 6 soluble proteins. Forty-three patients were recruited. Two SNPs in the vascular endothelial growth factor receptor 3 (VEGFR-3) gene predicted lower overall survival: rs307826 with hazard ratio (HR) 3.67 (confidence interval [CI] 95%, 1.35-10.00) and rs307821 with HR 3.84 (CI 95%, 1.47-10.0). Interleukin-6 was associated with increased mortality: HR 1.06 (CI 95%, 1.01-1.12), and osteopontin was associated with shorter PFS: HR 1.087 (1.01-1.16), independently of Ki-67. Furthermore, levels of osteopontin remained higher at the end of the study in patients considered non-responders: 38.5 ng/mL vs. responders: 18.7 ng/mL, p-value=0.039. Dynamic upward variations were also observed with respect to IL-8 levels in sunitinib-refractory individuals: 28.5 pg/mL at baseline vs. 38.3 pg/mL at 3 months, p-value=0.024. In conclusion, two VEGFR-3 SNPs as well as various serum biomarkers were associated with diverse clinical outcomes in patients with well-differentiated pancreatic neuroendocrine tumors treated with sunitinib.

## INTRODUCTION

Pancreatic neuroendocrine tumors (PNETs) represent a heterogeneous group of neoplasms arising from pancreatic islets, with an incidence of <2 per 100 000 persons per year [1]. In the case of well-differentiated (G1/2), metastatic PNETs, evolution is generally indolent compared to adenocarcinoma of the pancreas [2]. One of its most salient biological traits is its extraordinary vascularization, which is associated with the expression of multiple proangiogenic molecules, such as *vascular endothelial growth factor receptors* (VEGFR) and *platelet-derived growth factor* (PDGF) or *fibroblast growth factors* (FGFs) [3]. The overexpression or activation of proangiogenic pathways (e.g., upregulation of hypoxia-response transcription factors, genes of cellular response to hypoxia, the VEGF/VEGFR axis and crosstalk between pericytes and endothelial cells involving VEGF and PDGF) promotes growth in PNETs by directly upregulating angiogenesis, in addition to other indirect mechanisms [4, 5]. Additionally, the *phosphatidilinositol-3-kinase* (PI3K)-AKT-mTOR (*mammalian target of rapamycin*) pathway acts as the central hub for several cell programs in PNETs, including the participation in complex crosstalk regulating VEGF synthesis [6]. Indeed, the use of mTOR inhibitors is able to prolong progression-free survival (PFS) in advanced PNETs [7].

Among the angiogenesis inhibitors tested in PNETs, sunitinib malate (Sutent, Pfizer) is an oral, multitargeted tyrosine kinase inhibitor (TKI) primarily targeting VEGFR-1, VEGFR-2, VEGFR-3, RET,*stem-cell factor receptor* (c-kit), and PDGFR-α/β. In the SUN-1111 study, a phase III clinical trial in patients with well-differentiated PNETs in progression, sunitinib 37.5 mg/day increased PFS compared to placebo (11.4 *versus* 5.5 months; hazard ratio [HR], 0.42; p<0.001), with an objective response rate of 9.3% and a favorable safety profile [8]. Certain germline single-nucleotide polymorphisms (SNPs) in VEGFR-3, VEGFA, interleukin-8 (IL-8), FGFR2, c-KIT, or PDGFB have been correlated with clinical outcomes in subjects receiving sunitinib [9, 10]. In addition, various SNPs in genes that participate in metabolic pathways, efflux transporters and cell detoxification, such as *ATP-binding cassette sub-family B member 1* (ABCB1), *Nuclear Receptor Subfamily 1 Group I Member 2* (NR1I2) and *cytochrome P450* (CYP3A5), might be associated with its therapeutic efficacy or toxicity [9, 11]. However, no subgroup effect was noted in the SUN-1111 trial and, consequently, to date, no validated biomarker has been able to be incorporated into clinical practice for use in PNETs.

Furthermore, this high angiogenic profile is associated with the secretion of a repertoire of soluble molecules (e.g., proangiogenic cytokines and growth factors) that might be predictive biomarkers involved in the development of resistance to antiangiogenic drugs [12, 13].

In this situation, we have designed a prospective, multicenter study (Search activity in the laboratory for sunitinib, SALSUN) to identify biomarkers and SNPs involved in the efficacy or tolerability of sunitinib in patients with well-differentiated PNETs with metastatic disease in progression.

## RESULTS

### Patients

Forty-three patients treated between November 2012 and February 2015 were recruited; the follow-up database was closed in October 2017. All were evaluable for efficacy and safety endpoints, although SNPs were not available for two patients. Patients’ baseline clinical characteristics are summarized in Table 1. In particular, sunitinib was administered as first-line treatment in 41.9% (n=18), as second-line in 34.9% (n=15), and as successive lines in the remaining subjects. The starting dose of sunitinib was 37.5 mg per day. Subjects remained on treatment with sunitinib for a median of 6.3 months (range, 0.2-29); at six months, 37% of them (n=16) had discontinued treatment: 56.3% (n=9) due to progression, 18.8% (n=3) because of toxicity, and the remaining 25.0% (n=4) for another reason. The median follow-up was 51.3 months (95% confidence Interval [CI]), 44.2-58.3). In the population evaluable for efficacy endpoints (n=43), 40 progression events (93%) were detected and median PFS was 12.0 months (95% CI, 7.2-16.7). Moreover, 40 deaths (93%) were recorded with a median OS of 53.5 months (95% CI, 45.4-61.6).

**Table 1:** Baseline characteristics of the patients

	Patients, N=43
**Sex (female)**	20 (46.5%)
**Age (median, range)**	56 (28-77)^*^
**ECOG-PS**	
**0**	24 (55.8%)
**1**	19 (44.2%)
**Location of the tumor**	
**Head**	12 (27.9%)
**Head and body**	1 (2.3%)
**Tail**	16 (37.2%)
**Tail and body**	4 (9.3%)
**Body**	2 (4.6%)
**NR**	6 (13.9%)
**Tumor stage**	
**Locally advanced**	1 (2.3%)
**Metastatic**	42 (97.7%)
**Number of metastatic locations**	
**1**	22 (51.1%)
**2**	15 (34.8%)
**3**	1 (2.3%)
**4**	3 (6.9%)
**NR**	2 (4.6%)
**Metastatic locations**	
**Lung**	1 (2.3%)
**Nodes**	13 (30.2%)
**Bone**	4 (9.3%)
**Liver**	40 (93.0%)
**Others**	5 (11.6%)
**Functioning tumor**	6 (13.9%)
**Concurrent SSA**	19 (44.1%)
**Prior treatments**	
**None**	18 (41.8%)
**SSA**	13 (30.2%)
**Chemotherapy**	6 (13.9%)
**SSA and chemotherapy**	5 (11.6%)
**SSA and radiotherapy**	1 (2.3%)
**Ki-67 index**	
**<3**	9 (20.9%)
**3-10**	16 (37.2%)
**11-20**	12 (27.9%)
**NR**	6 (13.9%)

The percentage refers to columns. ^*^The datum for age expresses the median and range. Abbreviations: N = sample size, NR = not reported, ECOG-PS = Eastern Cooperative Group Performance Status, SSA = somatostatin analogue.

As for the objective response rate at 6 months (n=41), no patient attained a full response; 6 (14.6%) exhibited partial response; 30 (73.2%) displayed stable disease, and 5 individuals (12.2%) progressed. Consequently, the rate of clinical benefit was 87.8% (n=36). In the population suitable for analysis for safety endpoints (n=43), the most common grade 1-4 toxicities were asthenia (n=31; 72%), diarrhea (n=22; 51%), hypothyroidism (n=9; 21%), neutropenia (n=18; 42%), hand-foot syndrome (n=13; 30%), arterial hypertension (n=13; 30%), and thrombocytopenia (n=8; 19%). There was only a single case of grade 4 toxicity (diarrhea). Dose reduction due to toxicity was required in 16 patients (37%). The breakdown of the severity of these toxicities is shown in Supplementary Table 1.

Of the 43 evaluable patients, a sample for genotyping SNPs was available for 41 (1 patient was randomly lost to follow-up and another sample was hemolyzed on receipt). Supplementary Table 2 presents the SNPs analyzed and allele frequencies, all of which were compatible with the Hardy and Weinberg equilibrium, *p*>0.05. As for circulating biomarkers, blood samples for baseline determination were received for 36 of 43 subjects (83%); in the remaining cases (n=7), material was not available either because there was insufficient blood or tubes were missing. At three months, 31 blood samples were available (two individuals died before sampling; three were missing for purely administrative reasons). At the end of the study, we had four additional drop-outs (two due to severe clinical impairment, two missing values for administrative reasons).

### Effect of polymorphisms on the rate of clinical benefit, PFS, and OS

**Figure 1:**
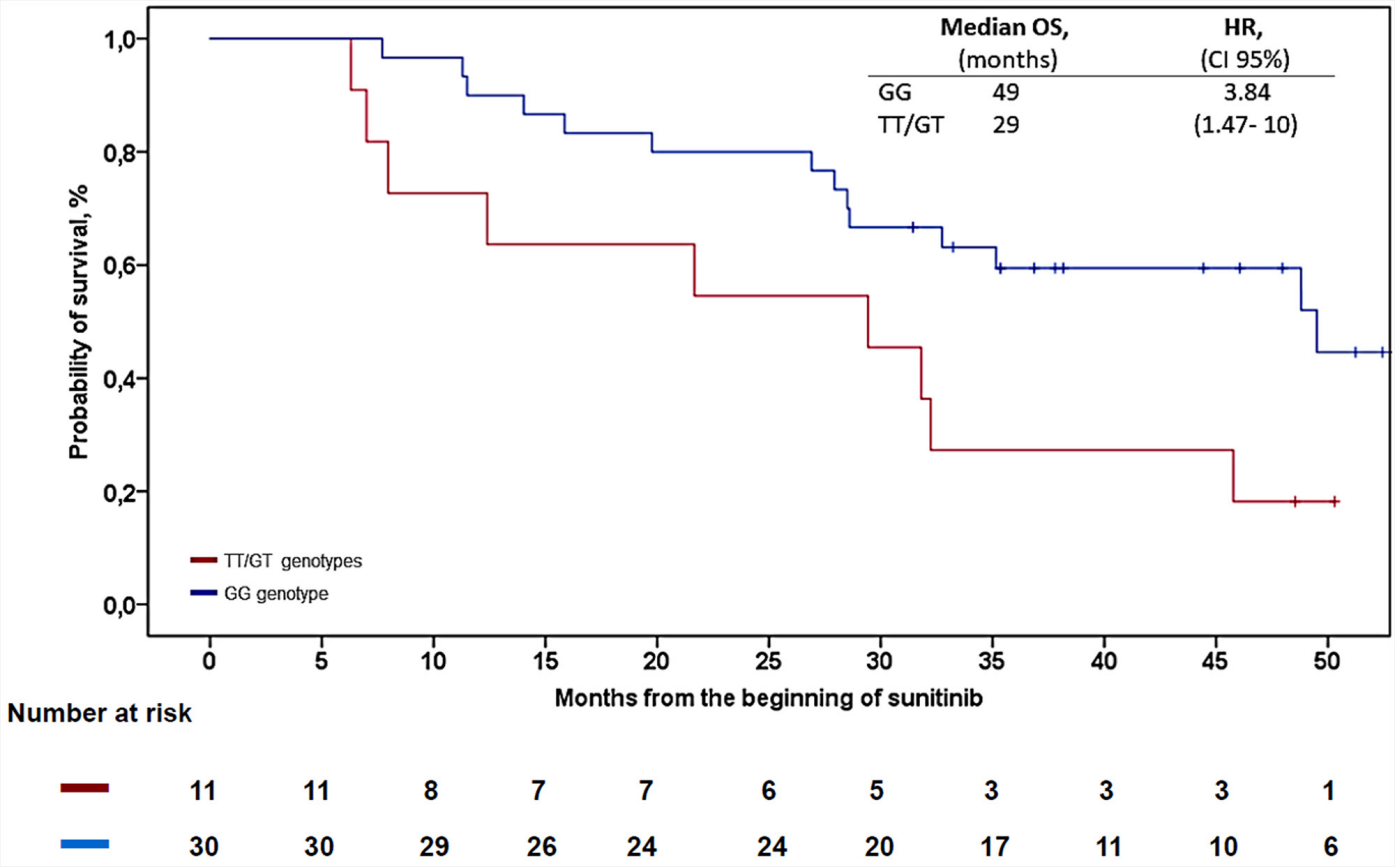
Kaplan-Meier analysis for OS in patients with rs307821 Abbreviations: OS = overall survival, HR = hazard ratio, CI = confidence interval.

Table 2 displays the results of Cox’s PH regression for survival endpoints (PFS/OS) and binary logistic regression for clinical benefit. Two polymorphisms related to angiogenesis were significantly associated with OS: VEGFR-3 c.1480A>G (rs307826) with HR 3.67 (CI 95%, 1.35-10.00), and VEGFR-3 c.4202G>T (rs307821) with HR 3.84 (CI 95%, 1.47-10). The wild-type genotype of VEGFR-3 was associated with increased median overall survival (OS) (49 months; 95% CI, 28-71) compared to the 29 months, (CI 95% CI, 8-50) for rs307821 GT/TT allele variants (Log Rank, *p*=0.027) (see Figure 1). None of the genotypes analyzed were associated with the probability of objective response, clinical benefit or PFS (Table 2). Insofar as safety data are concerned, the only predictive factor associated with a higher percentage of dose reductions was the VEGFR-3 rs307826 SNP. Thus, the dose was lowered in the first 6 months in 21% of the homozygous wild-type subjects *vs.* 59% in patients with the other genotypes; odds ratio (OR) 5.33 (CI 95%, 1.20-23.65), *p*=0.027 (see Table 3). Patients with variant VEGFR-3 alleles (rs307821) had a higher rate of hypothyroidism: 45% vs. 36%, OR 5.41 (CI 95%, 1.10-26.46), *p*=0.041. No other SNP was found to be associated with toxicity (of any type) or with antiangiogenic side effects (Table 3). No differences were detected in treatment duration based on the SNPs analyzed. None of these tests was significant after applying Holm-Bonferroni correction.

**Table 2:** Effect of polymorphisms on clinical benefit rate, PFS, and OS

	Reference SNP ID	Clinical benefit		PFS		OS	
		OR (95% CI)	p-value	HR (95%, CI)	p-value	HR (95%, CI)	p-value
**VEGFR3**	rs307826	1.43 (0.12-17.52)	0.782	1.31 (0.64-2.67)	0.464	3.67 (1.35-10)	**0.010**
	rs307821	NC	0.999	1.04 (0.50-2.18)	0.917	3.84 (1.47- 10)	**0.005**
**VEGFA**	rs1570360	0.87 (0.07-10.93)	0.912	1.04 (0.49-2.50)	0.926	2.38 (0.83-7.14)	0.104
	rs2010963	1.20 (0.09-15.09)	0.889	0.90 (0.44-1.85)	0.784	0.82 (0.32-2.12)	0.685
	rs699947	0.21 (0.02-2.68)	0.230	1.14 (0.52-2.49)	0.745	1.02 (0.37-2.81)	0.962
**IL-8**	rs4073	0.87 (0.07-10.93)	0.912	1.04 (0.49-2.18)	0.926	2.38 (0.83-7.14)	0.104
**FGFR2**	rs2981582	NC	0.999	1.05 (0.46-2.40)	0.905	1.70 (0.58-4.98)	0.333
**NR1|2**	rs3814055	0.62 (0.05-7.57)	0.706	1.43 (0.70-2.91)	0.329	1.41 (0.57-3.51)	0.461
**c-KIT**	rs6554199	1.20 (0.09-15.09)	0.889	0.90 (0.44-1.85)	0.784	0.82 (0.32-2.12)	0.685
**PDGFB**	rs130650	NC	0.999	0.54 (0.25-1.15)	0.107	0.55 (0.21-1.40)	0.206
**ABCB1**	rs1045642	NC	0.999	1.85 (0.78-4.34)	0.162	2.12 (0.70-6.66)	0.174
	rs1128503	1.51 (0.12-19.52)	0.750	0.92 (0.41-2.08)	0.842	0.78 (0.28-2.19)	0.642
	rs2032582	1.24 (0.10-15.51)	0.869	0.90 (0.41-1.96)	0.790	0.72 (0.26-2.02)	0.536
**CYP3A5**	rs776746	NC	0.999	0.81 (0.34-1.92)	0.625	0.57 (0.16-2.0)	0.378

The comparison between homozygous wild-type genotype *vs.* other genotypes (used as a reference) are shown. Abbreviations: NC = not computable, PFS = progression-free survival, HR = hazard ratio, CI = confidence interval, OR = odds ratio, OS = overall survival. An OR<1 indicates that clinical benefit is less likely in subjects with the SNP. *P*-values are derived from bivariate binary logical regression for clinical benefit rate, and Cox proportional hazards regression for survival endpoints; adjusted by Ki67 index. Tests were not corrected for multiple comparisons. The clinical benefit rate is the sum of complete and partial responses and stable disease at 6 months.

**Table 3:** Genetic factors related to dose reductions and adverse effects

	Reference SNP ID	Dose reductions due to toxicity		Hypertension		Hand-foot syndrome		Hypothyroidism		Mucositis		Diarrhea	
		OR (95%, CI)	P-value	OR (95%, CI)	P-value	OR (95%, CI)	P-value	OR (95%, CI)	P-value	OR (95%, CI)	P-value	OR (95%, CI)	P-value
**VEGFR3**	rs307826	0.15 (0.20-0.82)	0.040	4.40 (1.05-18.35)	0.067	2.62 (0.65-10.58)	0.277	3.19 (0.69-14.66)	0.23	1.70 (0.46-6.28)	0.512	1.93 (0.51-7.31)	0.509
	rs307821	0.25 (0.04-1.37)	0.151	1.57 (0.36-6.84)	0.701	1.57 (0.36-6.84)	0.701	5.41 (1.10-26.46)	**0.041**	1.25 (0.31-5.03)	1	1.75 (0.42-7.25)	0.499
**VEGFA**	rs1570360	0.77 (0.21-2.79)	0.750	0.85 (0.21-3.37)	1	0.52 (0.13-2.06)	0.484	0.22 (0.04-1.09)	0.118	0.34 (0.09-1.25)	0.120	0.55 (0.15-1.99)	0.522
	rs2010963	0.56 (0.15-2.01)	0.517	1.62 (0.39-6.62)	0.728	1.62 (0.39-6.62)	0.728	1.55 (0.32-7.34)	0.711	1.02 (0.28-3.60)	1	1.05 (0.30-3.65)	1
	rs699947	1.16 (0.30-4.60)	0.824	1.35 (0.29-6.20)	1	0.76 (0.17-3.24)	0.999	0.78 (0.16-3.82)	1	2.80 (0.62-12.4)	0.296	1.23 (0.31-4.73)	1
**IL-8**	rs4073	0.96 (0.25-3.58)	1	0.85 (0.21-3.37)	1	0.52 (0.13-2.06)	0.489	0.22 (0.04-1.09)	0.118	0.36 (0.10-1.33)	0.194	0.96 (0.28-3.30)	1
**FGFR2**	rs2981582	2.43 (0.54-10.89)	0.305	2.63 (0.48-14.41)	0.295	0.76 (0.17-3.24)	0.999	0.78 (0.16-3.82)	1	0.98 (0.23-3.86)	1	0.46 (0.11-1.92)	0.324
**NR1|2**	rs3814055	0.96 (0.26-3.52)	1	0.80 (0.20-3.12)	1	0.29 (0.07-1.23)	0.162	0.29 (0.07-1.23)	0.162	1.17 (0.32-4.24)	1	2.03 (0.56-7.31)	0.442
**c-KIT**	rs6554199	1.77 (0.50-6.37)	0.377	1.62 (0.39-6.62)	0.728	1.62 (0.39-6.62)	0.728	1.55 (0.32-7.34)	0.711	1.02 (0.28-3.60)	1	1.05 (0.30-3.70)	1
**PDGFB**	rs130650	0.64 (0.17-2.47)	0.525	0.62 (0.13-2.87)	0.718	1.11 (0.26-4.66)	1	2.04 (0.44-9.38)	0.428	0.51 (0.12-2.06)	0.498	1.60 (0.41-6.118)	0.524
**ABCB1**	rs1045642	1.64 (0.35-7.69)	0.711	4.00 (0.73-21.83)	0.150	1.17 (0.28-4.92)	1	1.27 (0.21-7.45)	1	1.41 (0.36-5.44)	0.739	0.20 (0.03-1.12)	0.073
	rs1128503	0.91 (0.21-3.99)	1	0.31 (0.07-1.43)	0.231	2.10 (0.37-11.85)	0.462	1.27 (0.21-7.45)	1	3.73 (0.67-20.6)	0.152	2.12 (0.49-9.19)	0.464
	rs2032582	0.66 (0.16-2.74)	0.718	0.22 (0.05-1.01)	0.061	1.26 (0.26-5.92)	1	0.72 (0.14-3.61)	1	4.50 (0.82-24.6)	0.085	1.60 (0.39-6.50)	0.722
**CYP3A5**	rs776746	1.25 (0.23-6.56)	1	1.91 (0.35-10.32)	0.653	0.88 (0.14-5.33)	1	1.42 (0.22-9.00)	1	1.09 (0.17-5.88)	1	0.58 (0.11-3.04)	0.682

The comparison between the dominant homozygous model and remaining genotypes (used as reference) is presented. The homozygous wild-type genotype is compared to the rest, referencing toxicities of any grade. *P*-values have been established based on Fisher’s exact tests for probability. Abbreviations: OR = odds ratio, CI = confidence interval.

### Effect of circulating biomarkers on response and survival endpoints

**Figure 2:**
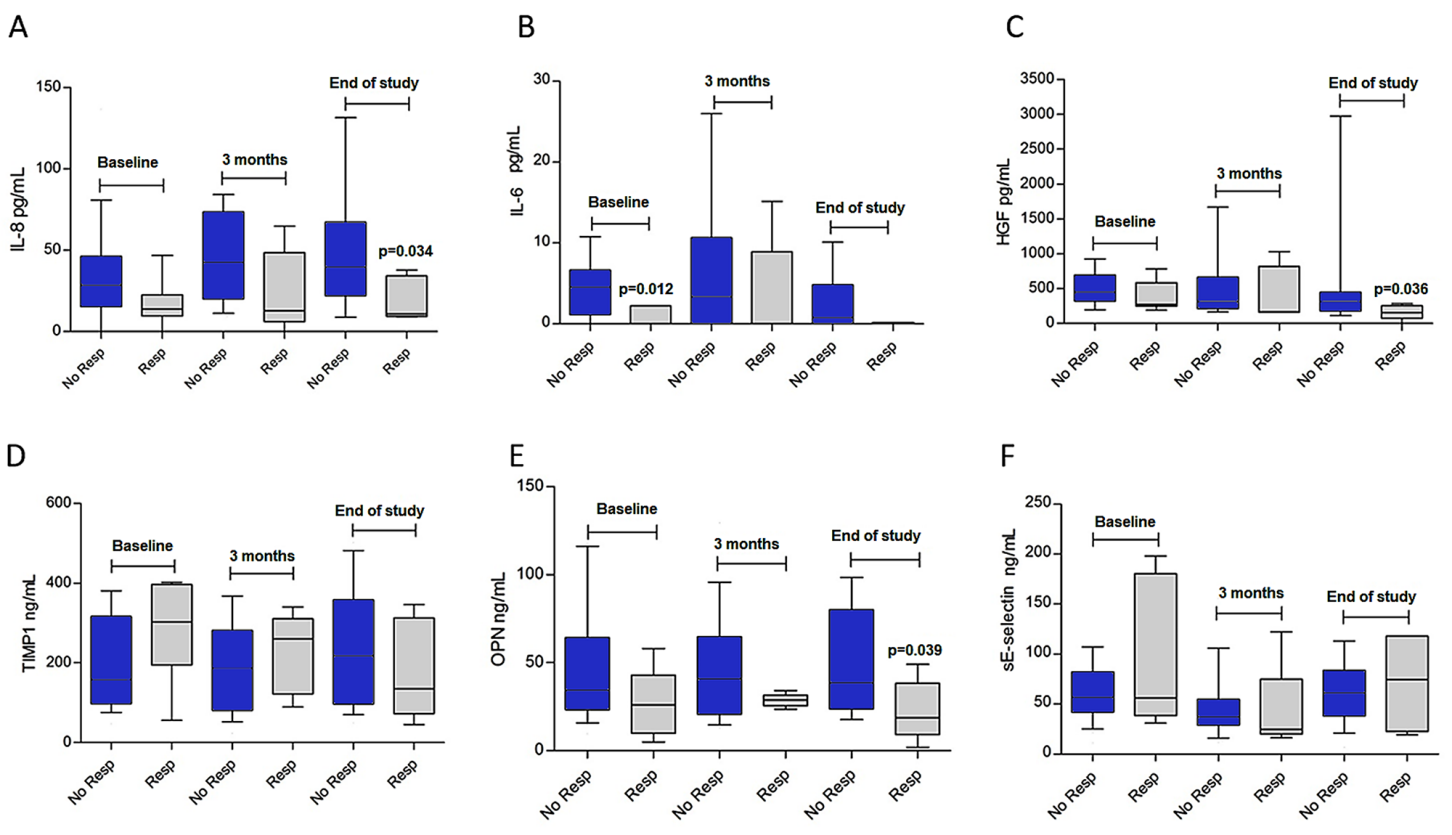
Levels of circulating biomarkers at baseline, three months, and at the end of follow-up **(A)** Interleukin-8 (IL-8); **(B)** interleukin-6 (IL-6); **(C)** hepatocyte growth factor (HGF); **(D)** tissue inhibitor of metalloproteinase-1 (TIMP-1); **(E)** osteopontin (OPN) and **(F)** sE-selectin*P*-values are derived from U-Mann Whitney tests to compare differences between subjects who responded (Resp.) and those who did not respond to treatment (No resp.). Blood samples were available for baseline determinations in 36 patients (30 non-responders, 6 responders); at three months in 31 patients (26 non-responders, 5 responders); and 27 subjects at the end of the study (22 non-responders, 5 responders). Responders were those with complete or partial response; non-responders are those with tumor progression or stabilization at 3 months.

Possible temporary, sunitinib-sensitivity dependent variations were analyzed (see Table 4). All *p*-values were Holm-Bonferroni corrected. A significant increase was noted in IL-8 levels in subjects with progressive disease at 3 months: 28.5 pg/mL (interquartile range [IQR], 30.9) at baseline *vs.* 38.3 pg/mL (IQR, 41.0), *p*-value=0.024. In contrast, responders did not exhibit dynamic changes: 13.7 pg/mL (IQR, 1.86) at baseline *vs.*12.6 pg/mL (IQR, 20.28) at 3 months, *p*-value=0.345. Similarly, baseline levels of sE-selectin decreased at 3 months in both responders and non-responders (*p*<0.001), but then increased at the end of the study (see Table 4). We observed other tendencies but dynamic differences could not be statistically confirmed for the remaining biomarkers during follow-up (see Table 4); for instance, TIMP-1 levels were 30.5% lower in subjects with response *versus* 10.1% higher in patients who did not achieve response, *p*-value=0.21 (Figure 2D).

**Table 4:** Levels of biomarkers based on sensitivity to sunitinib

		Baseline	3 months	End of study	*p*-value (adjusted^‡^)^§^	*p*-value (adjusted^‡^)^§§^
***IL-8,*** ***pg/mL***	Non-responders	28.56 (30.93)	38.37 (41.04)	39.45 (42.72)	**0.002 (0.024)**	**0.001 (0.012)**
	Responders	13.73 (1.86)	12.63 (20.28)	10.74 (21.00)	0.345 (1)	0.224 (1)
***IL-6,*** ***pg/mL***	Non-responders	4.55 (5.1)	3.33 (8.64)	0.75 (4.74)	0.180 (1)	0.064 (0.512)
	Responders	0.00 (2.22)	0.00 (2.73)	0.00 (0.00)	0.296 (1)	0.317 (1)
***HGF,*** ***pg/mL***	Non-responders	446.42 (346.38)	309.39 (450.09)	315.45 (266.22)	0.107 (0.967)	**0.009 (0.099)**
	Responders	273.14 (254.01)	162.15 (22.02)	154.68 (74.40)	0.345 (1)	0.080 (0.480)
***TIMP-1, ng/mL***	Non-responders	158.87 (215.97)	186.08 (193.94)	218.40 (254.65)	0.056 (0.560)	**0.043 (0.430)**
	Responders	303.20 (154.32)	260.57 (125.60)	134.65 (179.40)	0.136 (1)	0.455 (0.910)
***OPN,*** ***ng/mL***	Non-responders	34.36 (36.88)	40.90 (40.14)	38.57 (53.58)	0.253 (1)	0.654 (0.654)
	Responders	25.88 (26.25)	28.87 (1.28)	18.78 (11.32)	0.893 (1)	0.345 (0.690)
***sE-selectin, ng/mL***	Non-responders	56.83 (40.14)	36.95 (25.78)	60.92 (36.37)	0.003 (0.033)	0.064 (0.576)
	Responders	56.03 (117.02)	24.52 (3.52)	64.09 (79.01)	0.068 (0.612)	0.068 (0.476)

Responders are those with complete or partial response; non-responders are those with tumor progression or stabilization at 6 months. Paired data were compared with the Wilcoxon Signed-Rank Test. The numbers for the “baseline”, “3 months” and “End of Study” columns represent the median, and the interquartile range (in brackets). ^‡^Raw p-values and p-values adjusted using the Bonferroni-Holm method (in brackets) for multiple comparisons are displayed. ^§^Comparison between baseline and 3-month measures. ^§§^Comparison between baseline and exit measures. Abbreviations: IL-6 = interleukin-6, IL-8 = interleukin-8, HGF = hepatocyte growth factor, TIMP-1 = tissue inhibitor of metalloproteinase-1, and OPN = osteopontin.

Median levels of proinflammatory cytokines (IL-6 and IL-8), HGF, and OPN tended to be lower at the end of the study in individuals exhibiting tumor response in comparison with subjects with no response (see Figure 2A, 2B, 2C & 2E). However, after adjusting for the family-wise error associated with multiple comparisons, none were significant (*p*-value >0.05 for all). In the case of IL-6, circulating levels remained all but undetectable in patients displaying tumor response throughout the entire follow-up period, with a baseline median of IL-6: 0 (IQR, 2.22) *vs.* 4.5 pg/mL (IQR, 5.1), *p*-value=0.072 (see Figure 2D). However, absolute levels of TIMP-1 and sE-Selectin were not significantly different at any point during follow-up for subjects with or without response (see Figure 2A, 2B, 2C and 2D).

Finally, we analyzed whether the dynamic changes (reduction *vs.* increase) in each of the soluble markers exerted any effect on OS-related endpoints in addition to the Ki-67 index (see Supplementary Tables 4 & 6). Multiple comparison adjustments were not applied for time-to-event analyses, due to their exploratory purpose. Only IL-6 levels (pg/mL, continuous time-dependent variable) were significantly associated with increased mortality, with a HR of 1.068 (CI 95%, 1.013-1.126), *p*-value=0.013. The effect was similar when restricted to baseline measures with HR 1.184 (CI 95%, 1.061-1.321), *p*-value=0.018. Similarly, OPN levels were associated with lower PFS, independent of the Ki67% index, with HR 1.087 (1.011-1.169), *p*=0.023. Ki-67 expression was significantly associated with shorter both PFS and OS, as expected (see Supplementary Tables 3 & 5).

## DISCUSSION

This is a prospective, multicenter study of the effect of 14 SNPs and 6 circulating biomarkers on safety and efficacy endpoints in patients with low-grade PNETs (G1/G2, WHO 2010) who received sunitinib. Basically, the work explores how these profiles modify the prognosis, and potentially influence differential response to this drug.

The reason for conducting this analysis was that certain key aspects of optimal selection of patients with a greater benefit-risk ratio have yet to be formally elucidated. In particular, the pivotal trial (SUN-1111) failed to report the analysis of subgroups based on genetic profiles or in-depth analyses of the evolution of circulating biomarkers according to response to sunitinib, which implied a lack of critical information both regarding key pharmacogenomic aspects of the therapy, as well as about the influence of tumor markers on tumor evolution [8].

The VEGFR-3 axis, through various signalling pathways, has a critical role in cancer progression by regulating different cellular functions such as angiogenesis, tumor growth, proliferation and chemotherapy resistance [14]. Neuroendocrine tumors are also highly dependent on VEGFR-3 signalling, which, in turn, is one of the most important targets of sunitinib [8]. Here, we have found that two missense mutations in VEGFR-3 (rs307826 and rs307821), present respectively in 34% and 26% of the participants in this study, are associated with decreased OS. VEGFR-3 c.1480A>G (rs307826) involves the exchange of a residue threonine for alanine, which is associated with a deficit of protein expression [15]. The modification of VEGFR-3 c.4202G>T (rs307821) is likely to have functional implications [9]. In both cases, these changes have previously been linked to decreased PFS in patients with PNETs, treated with pazopanib [16] or other drugs [17]. This has generally been attributed to resistance to tyrosine-kinase inhibitors [16, 18, 19].

Thus, Beuselinck’s renal cancer series reported that rs307826 affected survival outcomes with a median of 31 months of OS for genotype AA vs. 22 months for AG/GG variants (p*=*0.013) [18]. In contrast, García-Donas et al. reported a series of 89 kidney cancer patients treated with sunitinib without any association with OS [9]. Our data also suggest that a SNP in the VEGFR-3 gene (rs307821) increases the likelihood of developing hypothyroidism in patients treated with sunitinib. The biological mechanism of this correlation is not clear, although a tentative explanation could be the increased vulnerability of the microvasculature of the thyroid gland to VEGFR2 inhibition when the VEGFR-3 signaling pathway is constitutively affected [20].

In line with previous research [21], our data also suggest how baseline levels and dynamic changes of soluble molecules implicated in alternative proangiogenic pathways, proinflammatory mechanisms, cell adhesion or migration (mainly IL-6, IL-8, and sE-selectin) vary differently according to the objective response to sunitinib. One of the most surprising outcomes is the verification that objective responses to sunitinib are accompanied by significantly lower or even undetectable levels of the proangiogenic cytokines IL-8 and IL-6, in line with data obtained in other cancers. In fact, IL-8 is a proangiogenic factor produced by tumor-infiltrating macrophages and other tissues, whose levels rise with exposure to sunitinib in several cancers, including PNETs [13, 22], which would appear to be part of a mechanism of drug resistance [23].

We have also found moderate statistical evidence that elevated OPN levels shortened PFS in this series. OPN is an extracellular protein that mediates interactions with integrins and components of the extracellular matrix, enhances angiogenesis by activating the PI3K/AKT and ERK pathways [24, 25], which comprises a probable mechanism of acquired active resistance to antiangiogenics in several tumors [26].

Interestingly, we found that IL-6 constitutes an adverse prognostic factor [HR 1.068 (1.013-1.126)], independent of Ki67 index, which may actually help in the prognostic stratification of these tumors [27]. Our data support the finding that dynamic changes in other soluble molecules, such as sE-selectin, would also correlate with acquired resistance to sunitinib in PNETs and could be potential surrogate biomarkers of clinical benefit associated with antiangiogenic therapy in this neoplasm. sE-selectin plays a role in angiogenesis as an adhesion molecule and its effect is capable of modulating response to antiangiogenics, possibly through endothelial cell recruitment, and is a biomarker of VEGF-inhibiting therapies [28, 29].

Our data have limitations, and the reader must be aware that the findings of this hypothesis-generating study call for prospective validation with a larger sample. The absence of a comparator group precludes the assessment of interactions between SNPs and treatment effects. With the limited sample size, this study is underpowered and can hence best be considered as hypothesis-generating. As an example, the power to determine the increase of Il-8 between baseline and 3 months in the non-responders group by Wilcoxon Signed-Ranked Test is about 43%. When correction for multiple testing is applied for all correlations with the SNPs, none remain significant. However, the Holm–Bonferroni procedure is usually considered a relatively conservative method. As with other studies of SNPs, no pharmacokinetic data are available for sunitinib (or its metabolites) or for VEGF/VEGFR levels to correlate with each one of the genetic variants, though this endpoint would have been compelling. Moreover, concurrent use of SSA in 44% of patients may introduce an element of uncertainty into the assessment of PFS in this series [30]. Finally, the tumor itself has not been genotyped, which could certainly be informative, although other authors have found a high degree of concordance with the genetic profile of the germ line [31].

In conclusion, the exploratory SALSUN study points to an association between VEGFR-3 SNPs (rs307826 and rs307821) and various serum biomarkers (IL-6 and OPN) involved in alternative proangiogenic pathways and mechanisms of resistance, and clinical outcomes during the course of therapy with sunitinib, and might serve as surrogate endpoints in future clinical trials in PNETs. The search for individualized treatment algorithms should be promoted based on genetic variants and biomarkers associated with the signaling pathways of sunitinib targets.

## MATERIALS AND METHODS

### Patients

We have conducted a prospective phase IV study, in 11 Spanish hospitals. All adult patients (≥18 years) with a histological diagnosis of well- or moderately-differentiated (Ki-67 ≤20%) metastatic PNET, scheduled to begin treatment with sunitinib as per clinical practice at each center were consecutively enrolled. Other eligibility criteria included the presence of measurable tumor disease by *Response Evaluation Criteria In Solid Tumors* (RECIST) 1.1 criteria, with confirmed radiological progression in the previous 12 months and adequate hematological, hepatic, and renal function. Subjects had to have an *Eastern Cooperative Oncology Group* performance status (ECOG-PS) of 0-1 and could not have received prior treatment with mTOR inhibitors or antiangiogenic agents. Likewise, those individuals with symptomatic brain metastases, prior cardiac events or thromboembolic disease in the previous 12 months were excluded. The study was approved by the Ethic Committee of the Salamanca University Hospital, and all participants provided written informed consent.

### Procedures

Treatment procedures and clinical decisions, including dose reduction policy or management of toxicities, were made by the investigators according to each center’s clinical practice. Histopathological and clinical data were collected with an electronic case report form and were regularly monitored externally to guarantee both the quality and safety of the process.

Molecular analyses were performed centrally at two different laboratories, blinded as to clinical or evolutionary data. Fourteen SNPs in 9 genes involved in the pharmacodynamic mechanism, metabolism and detoxification of sunitinib, were evaluated. These SNPs are located in genes that code for cytokines and tyrosine kinase receptors: VEGFR-3 (rs307826, rs307821), VEGFA (rs1570360; rs699947, rs2010963), interleukin-8 (IL-8) (rs4073), FGFR2 (rs2981582), c-KIT (rs6554199), PDGFB (rs3814055) [9, 10], as well as in genes that participate in cell detoxification and drug metabolism: ABCB1 (rs1045642, rs1128503, rs2032582), NR1I2 (rs3814055) and CYP3A5 (rs776746) [9, 10]. These SNPs were chosen following a review of the literature based on: the presence of prior evidence of their association with PFS/OS during treatment with sunitinib or pazopanib, that entailed a switch in the sequence of amino acids and that had minor allele frequencies ≥5% in previous studies [10–18]. With respect to the genes of angiogenesis, we focused on VEGFR-3 because the preclinical evidence suggests that it has a more active role than VEGFR-2 in the development of distant and lymphatic metastases [9]. DNA was isolated from peripheral blood by standard proteinase K and phenol-chloroform protocols. Blood was stored at -20°C until analysis. Genotyping was performed with Real-Time PCR and TaqMan SNP genotyping assays, using the StepOnePlus^®^ System to detect fluorescence and assign alleles (Applied Biosystems; Foster City, CA).

Simultaneously, hepatocyte growth factor (HGF), interleukin-6 (IL-6), IL-8, tissue inhibitor of metalloproteinase-1 (TIMP-1), sE-selectin, and osteopontin (OPN) levels were determined in patients’ blood samples. These molecules were chosen for their putative role in the angiogenic process and/or earlier evidence of sunitinib’s effect on their circulating levels [32]. Biomarker levels were quantified by the multiplex bead assays (Luminex xMAP) incorporated into the MILLIPLEX MAP kits and run on Luminex 200, according to the manufacturer’s instructions. Samples were drawn at baseline, three months and at the time of patient progression.

### Objectives & variables

We conducted a hypothesis-generating pilot study to correlate the presence of the afore-named SNPs and the trend of circulating biomarkers in responders and non-responders to sunitinib. Other exploratory objectives included establishing the correlation with clinical benefit and objective response rates, survival-based endpoints (PFS/OS), overall toxicity, class-specific toxicities, and dose reductions due to toxicity. OS was defined as the time elapsed between initiating therapy with sunitinib and the date of death due to any cause, whereas PFS was considered to be the time between treatment initiation until objective progression or demise. In any case, subjects with no event at the end of follow-up were censored. Objective response was evaluated locally by the researchers using RECIST 1.1 every 6 months until progression or withdrawal from the study [33]. Response was not centrally assessed. The rate of clinical benefit was defined as the sum of evaluations with complete or partial response, and stable disease. Adverse events were graded as per the *Common Terminology Criteria for Adverse Events* (CTCAE), version 3.0 [34]. Since the genotypes identified with homozygous variants were uncommon, we pooled these cases with heterozygous genotypes in the survival analysis. Tumors have been graded by Ki67 index values according to the 2010 WHO classification [35].

### Statistics

Hardy-Weinberg equilibrium (HWE) assumption was calculated by Chi-squared test for each SNP. The association between clinical and molecular variables was analyzed by crosstabs and Pearson’s chi-squared tests (χ2), while odds ratio (OR) and 95% confident intervals were calculated by logistic regression analyses. OS and PFS-related endpoints were estimated using the Kaplan-Meier method. To evaluate the effect of each SNP for each OS endpoint, Cox’s proportional hazards (PH) regression was used, adjusted by Ki67 index. Cytokines and serum biomarkers were treated as time-dependent variables [36]. Fisher’s exact test was applied to compare categorical variables. Statistical hypothesis testing for continuous variables was performed using the Mann-Whitney U test (due to the non-normality of these variables). Paired data were compared with the Wilcoxon Signed-Rank Test. All statistical assessments were two-sided and *p*-values<0.05 were deemed significant. Given the exploratory nature of the study in a field for which no published data were available during the design phase, no statistical tool was applied to calculate sample size. Furthermore, for the same reason, corrections for multiple comparison were performed only for the effect of circulating biomarkers on response. The SPSS version 19.0 statistical software package was used.

## SUPPLEMENTARY MATERIALS TABLES



## Abbreviations

(ABCB1): ATP-binding cassette sub-family B member 1
(CTCAE): Common Terminology Criteria for Adverse Events
(CYP3A5): Cytochrome P450
(ECOG-PS): Eastern Cooperative Oncology Group performance status
(FGFs): Fibroblast growth factors
(FGFR2): Fibroblast growth factor receptor 2
(HR): Hazard ratio
(HGF): Hepatocyte growth factor
(IL-6): Interleukin-6
(IL-8): Interleukin-8
(IQR): Interquartile range
mTOR: (mammalian target of rapamycin)
(NR1I2): Nuclear Receptor Subfamily 1 Group I Member 2
(OPN): Osteopontin
(OS): Overall survival
(PNETs): Pancreatic neuroendocrine tumors
(PDGFB): Platelet Derived Growth Factor Subunit B
(PI3K): Phosphatidilinositol-3-kinase
(PDGF): Platelet-derived growth factor
(RECIST): Response Evaluation Criteria In Solid Tumors 1.1 criteria
(SNPs): Single-nucleotide polymorphisms
(TIMP-1): Tissue inhibitor of metalloproteinase-1
(VEGFR): Vascular endothelial growth factor receptors
(VEGFA): Vascular Endothelial Growth Factor A
