# Supplementary material for: Biomarkers and polymorphisms in pancreatic neuroendocrine tumors treated with sunitinib
Source: Oncotarget. 2018 Dec 11;9(97):36894–905. doi: 10.18632/oncotarget.26380 (PMC6319342; doi:10.18632/oncotarget.26380)
Supplement: Supplementary file 1 [file oncotarget-09-36894-s001.pdf]

Biomarkers and polymorphisms in pancreatic neuroendocrine tumors treated with sunitinib

SUPPLEMENTARY MATERIALS

Supplementary Table 1: Summary of adverse events

TOXICITY		
	**Total**	**Grade 3-4**
**Anemia**	17%	0
**Neutropenia**	42%	22%
**Thrombocytopenia**	19%	5%
**Diarrhea**	51%	18%
**Stomatitis**	44%	5%
**Fatigue**	72%	16%
**Hand-foot syndrome**	30%	8%
**Hypertension**	30%	0
**Hypothyroidism**	21%	0
**Cardiotoxicity**	2%	0

Supplementary Table 2: Genotyping of polymorphisms and allele frequencies

	SNP	Variant	N	Homozygous wild-type	Heterozygous	Homozygous variant	MAF
**VEGFR3**	rs307826 A>G	T494A	41	27 (65.9%)	13 (31.7%)	1 (2.4%)	18.2%
	rs307821 G>T	R1324L	41	30 (73.2%)	10 (24.4%)	1 (2.4%)	14.6%
**VEGFA**	rs1570360 G>A	Promoter	41	16 (39.0%)	17 (41.5%)	8 (19.5%)	40.2%
	rs2010963 G>C	5′-UTR	41	17 (41.5%)	16 (39.0%)	8 (19.5%)	39.0%
	rs699947 A>C	Promoter	41	12 (29.3%)	20 (48.8%)	9 (22.0%)	46.3%
**IL-8**	rs4073 T>A	5′-UTR	41	16 (39.0%)	17 (41.5%)	8 (19.5%)	40.2%
**FGFR2**	rs2981582 C>T	Intron	41	12 (29.3%)	21 (51.2%)	8 (19.5%)	45.1%
**c-KIT**	rs6554199 T>G	Promoter	41	17 (41.5%)	16 (39.0%)	8 (19.5%)	39.0%
**NR1|2**	rs3814055 C>T	5′-UTR	41	19 (46.3%)	20 (48.8%)	2 (4.9%)	29.2%
**PDGFB**	rs130650 G>A	5′-UTR	41	28 (68.3%)	11 (26.8%)	2 (4.9%)	17.0%
**ABCB1**	rs1045642 C>T	I1145I	41	10 (24.4%)	16 (39.0%)	15 (36.6%)	43.9%
	rs1128503 C>T	G412G	41	11 (26.8%)	23 (56.1%)	7 (17.1%)	45.1%
	rs2032582 T>G	S893A	41	11 (26.8%)	23 (56.1%)	7 (17.1%)	45.1%
**CYP3A5**	rs776746 C>T	Splicing	41	32 (78.0%)	9 (22.0%)	0	10.9%

Abbreviations: MAF = Minor allele frequency, SNP = Single nucleotide polymorphism.

Supplementary Table 3: Cox proportional hazard regressions for PFS (univariate analyses) and time dependent biomarker levels as covariates, adjusted for Ki67

	Time-dependent biomarker		
	**Coeff.**	**HR (95% CI)**	***p*-value**
**IL-6 (pg/mL)**	0.0063	1.006 (0.973-1.040)	0.710
**IL-8 (pg/ml)**	-0.0001	0.999 (0.995-1.004)	0.957
**HGF (10 pg /mL)***	0.0027	1.002 (0.998-1.006)	0.165
**OPN (10 ng/mL)****	0.0840	1.087 (1.011-1.169)	**0.023**
**TIMP1 (10 ng/mL)****	-0.0052	0.994 (0.964-1.026)	0.740
**sE-selectin (ng/mL)**	-0.0031	0.996 (0.988-1.005)	0.492

Time to event in months.

* Cox model was computed with a change of scale: 10 pg/mL. Otherwise, results did not have enough precision.

** Cox model was computed with a change of scale: 10 pg/mL=10,000 ng/mL. Otherwise, results did not have enough precision.

Multiple comparison adjustment was not applied.

Abbreviations: Coeff = coefficient, HR = hazard ratio, CI = confidence interval, pg = picogram, ng = nanogram, mL = milliliter.

Supplementary Table 4: Cox proportional hazard regressions for OS (univariate analyses) and time dependent biomarker levels as covariates, adjusted for Ki67

	Time-dependent biomarker		
	**Coeff.**	**HR (95% CI)**	***p*-value**
**IL-6 (pg/mL)**	0.0667	1.069 (1.014-1.127)	**0.013**
**IL-8 (pg/ml)**	0.0014	1.001 (0.997-1.005)	0.474
**HGF (10 pg /mL)***	0.0012	1.001 (0.997-1.005)	0.537
**OPN (10 ng/mL)****	0.0606	1.062 (0.954-1.183)	0.269
**TIMP1 (10 ng/mL)****	0.0017	1.001 (0.964-1.040)	0.930
**sE-selectin (ng/mL)**	-0.0018	0.998 (0.986-1.010)	0.773

Time to event in months. Multiple comparison adjustment was not applied.

Abbreviations: Coeff = coefficient, HR = hazard ratio, CI = confidence interval, pg = picogram, ng = nanogram, mL = milliliter.

Supplementary Table 5: Cox proportional hazard regressions for PFS (multivariate analysis, backward stepwise selection of variables) and time dependent biomarker levels as covariates, adjusted for Ki67 index

		Coeff.	HR (95% CI)	*p*-value
**Initial model (all variables)**	KI67 index	0.08502	1.088 (1.007-1.176)	**0.031**
	IL-6 (pg/mL)	-0.01240	0.987 (0.949-1.027)	0.538
	IL-8 (pg/ml)	-0.00003	0.997 (0.995-1.004)	0.989
	HGF (10 pg /mL)*	0.00032	1.000 (0.994-1.005)	0.906
	OPN (10 ng/mL)**	0.08992	1.094 (0.988-1.211)	0.082
	TIMP1 (10 ng/mL)**	-0.00259	0.997 (0.963-1.032)	0.883
	sE-selectin (ng/mL)	-0.00257	0.997 (0.987-1.007)	0.604
**Final model**	KI67 index	0.08879	1.092 (1.017-1.174)	**0.015**
	OPN (10 ng/mL)**	0.08397	1.087 (1.011-1.169)	**0.023**

Backward stepwise selection of variables. Time to event in months. Multiple comparison adjustment was not applied.
Abbreviations: Coeff = coefficient, HR = hazard ratio, CI = confidence interval, pg = picogram, ng = nanogram, mL = milliliter.

Supplementary Table 6: Cox proportional hazard regressions for OS (multivariate analysis, backward stepwise selection of variables) and time dependent biomarker levels as covariates, adjusted for Ki67 index

		Coeff.	HR 95% CI	*p*-value
**Initial model (all variables)**	KI67 index	0.15894	1.172 (1.063-1.292)	**0.001**
	IL-6 (pg/mL)	0.06323	1.065 (1.004-1.129)	**0.033**
	IL-8 (pg/ml)	0.00268	1.002 (0.998-1.007)	0.222
	HGF (10 pg /mL)*	-0.00173	0.998 (0.991-1.004)	0.601
	OPN (10 ng/mL)**	0.07575	1.078 (0.878-1.325)	0.470
	TIMP1 (10 ng/mL)**	-0.01029	0.989 (0.940-1.041)	0.694
	sE-selectin (ng/mL)	0.00116	1.001 (0.986-1.016)	0.878
**Final model**	KI67 index	0.16161	1.175 (1.072-1.288)	**<0.001**
	IL-6 (pg/mL)	0.06670	1.068 (1.013-1.126)	**0.013**

Backward stepwise selection of variables. Time to event in months. Multiple comparison adjustment was not applied.
Abbreviations: Coeff = coefficient, HR = hazard ratio, CI = confidence interval, pg = picogram, ng = nanogram, mL = milliliter.
